# Elastisch-stabile intramedulläre Nagelung pertrochantärer Femurfrakturen im Kleinkindesalter (<6 bis 8 Jahre)

**DOI:** 10.1007/s00064-020-00696-2

**Published:** 2021-01-26

**Authors:** N. Kaiser, T. Slongo

**Affiliations:** Universitätsklinik für Kinderchirurgie, Inselspital Bern, Freiburgstr., 3010 Bern, Schweiz

**Keywords:** Proximale Femurfraktur, Titannagel, Minimal-invasive Versorgung, Trümmerfraktur, TEN, Proximal femur fracture, Titanium nail, Minimally invasive care, Comminuted fractures, TEN

## Abstract

**Operationsziel:**

Minimal-invasive, übungsstabile Versorgung von pertrochantären Femurfrakturen bei Kindern < 6 bis 8 Jahren mittels elastisch-stabiler intramedullärer Nagelung (ESIN).

**Indikationen:**

Proximale, pertrochantäre Femurfrakturen Delbet Typ IV bei Kindern < 6 Jahre.

**Kontraindikationen:**

Trümmerfrakturen, Schenkelhalsfrakturen.

**Operationstechnik:**

Durch Einbringen von insgesamt 3 (gelegentlich nur 2) im proximalen Drittel vorgebogenen, elastischen Titannägeln (TEN) retrograd in das Femur wird im proximalen Fragment eine stabile 3‑Punkt-Abstützung erreicht. Eine weitere Verbesserung der Stabilität kann durch die Applikation von Endcaps erreicht werden.

**Weiterbehandlung:**

Die Nachbehandlung erfolgt mittels Sohlenbelastung während 4 bis 5 Wochen. Röntgenkontrollen erfolgen direkt postoperativ sowie nach 4 bis 5 Wochen. Eine Sportunfähigkeit besteht für 3 Monate.

**Ergebnisse:**

In unserem Patientengut haben wir gute Erfahrungen mit dieser Technik bei den sehr seltenen pertrochantären Frakturen bei Kindern < 6 bis 8 Jahren. Mit minimal-invasivem Vorgehen kann eine übungsstabile Versorgung ohne Notwendigkeit eines Becken-Bein-Gipses erreicht werden.

## Abstract (en)

**Objective:**

Minimally invasive, sufficiently stable for movement and partial weight bearing, osteosythesis of pertrochanteric femoral fractures in children < 6–8 years using elastic, stable intramedullary nailing (ESIN).

**Indications:**

Proximal, pertrochanteric femoral fractures Delbet type IV in children < 6 years.

**Contraindications:**

Comminuted fractures, femoral neck fractures.

**Surgical technique:**

By inserting three elastic titanium nails (TEN), prebent in the proximal third, retrograde into the femur, a stable 3‑point support stabilizes the proximal fragment. For further improvement of stability, EndCaps can be used.

**Postoperative management:**

Partial weight bearing (sole-contact) for 4–5 weeks. X‑ray controls immediately after surgery and after 4–5 weeks. No sports for 3 months.

**Results:**

In our patient population we have good experience with this technique for very rare pertrochanteric fractures in children younger than 6–8 years. With minimally invasive access, exercise-stable administration can be achieved without a pelvic leg cast.

## Vorbemerkungen

Über die Inzidenz von Schenkelhals- (SH) und pertrochantären Frakturen des Femurs bestehen unterschiedliche Angaben je nachdem, in welcher Stellung ein Spital steht. Umfragen im Rahmen von Vorträgen bestätigen meist, dass selbst Traumazentren lediglich 1 bis 2, maximal 3 Frakturen pro Jahr sehen. Somit ist davon auszugehen, dass die Erfahrung in der Behandlung solcher Frakturen eher gering ist [3]. Unsere Klinik liegt im Einzugsgebiet mehrerer großer Skigebiete und deckt auch generell für die Frakturversorgung eher komplexer Frakturen beim Kind eine Region von ca. 1,3–1,4 Mio. Einwohnern ab. Die pertrochantären oder Delbet-Typ-4-Frakturen haben einen Anteil von nur 10 % an den Frakturen des proximalen Femurs und sind somit für den Behandler eine Seltenheit [6]. Wir behandeln in unserem Zentrum pro Jahr ca. 1000 Frakturen bei Kindern zwischen 1 und 16 Jahren. Hierbei handelt es sich bei im Schnitt 3 bis 4 Frakturen um solche des proximalen Femurs, und lediglich 1 dieser Frakturen ist eine pertrochantäre Fraktur. Pertrochantäre Femurfrakturen werden wie andere Frakturen dieser Region in der Regel mit einer offenen Reposition mit interner Fixierung (ORIF) mittels Platten oder Schraubenosteosynthese versorgt [3]. Jedoch erlaubt die extraartikuläre Lage dieser Frakturen und das damit verbundene, im Vergleich zu den Schenklhalsfrakturen praktisch fehlenden Risiko (0 %) für eine avaskuläre Nekrose (AVN) [4, 5] auch ein Vorgehen mittels geschlossener Reposition und Stabilisierung. Eine Option der Behandlung dieser Frakturen beim Kind < 6 bis 8 Jahre ist die Ruhigstellung im Becken-Bein-Gips während 4 bis 6 Wochen. Dislozierte Frakturen müssen vor einer Ruhigstellung reponiert werden. Diese Reposition kann akut, aber auch durch eine Extension während 1 bis 2 Wochen erreicht werden. Ein großer Nachteil der konservativen Behandlung sind die Notwendigkeit wöchentlicher radiologischer Kontrollen und die damit verbundene hohe Strahlenbelastung des Kindes sowie die völlige Immobilisation im Becken-Bein-Gips [1]. Eine alternative Behandlung dieser Frakturen ist die geschlossene Reposition und Osteosynthese mittels minimal-invasiver, elastisch-stabiler intramedullärer Nagelung (ESIN), durch die eine übungsstabile Versorgung dieser Frakturen auch beim Kind < 6 bis 8 Jahre erreicht werden kann. Die Technik der ESIN für Schaftfrakturen des Femurs, in den 1980er-Jahren durch die Nancy-Gruppe um Métaizeau und Prévot erstmals publiziert [2], ist heute eine der Standardtechniken für diese Brüche. Für den in dieser Technik geübten Operateur ist jedoch unter der Beachtung einiger Besonderheiten auch eine Ausweitung der Indikation auf die proximalen, metaphysären Brüche des Femurs, die pertrochantären Frakturen, möglich. Aus Stabilitätsgründen sollte diese Versorgungstechnik nicht bei größeren/älteren Kindern angewendet werden.

## Operationsprinzip und -ziel

Minimal-invasive, übungsstabile Versorgung von extraartikulären, pertrochantären Femurfrakturen bei Kindern < 6 bis 8 Jahre durch elastisch-stabile intramedulläre Nagelung (ESIN). Die Stabilität wird in dieser Situation durch das Aufspannen von 3 vorgebogenen elastischen Titannägeln (TEN) erreicht.

## Vorteile

- Minimal-invasive Methode, besonders gut geeignet für diese Altersgruppe, in der andere Operationsverfahren eher zu invasiv sind
- In aller Regel übungsstabile Versorgung ohne Notwendigkeit eines Becken-Bein-Gipses
- Geschlossene Reposition in den meisten Fällen möglich

## Nachteile

- Technisch anspruchsvoll
- Entfernung des Osteosynthesematerials empfohlen

## Indikationen

- Proximale, pertrochantäre Femurfrakturen Delbet Typ 4, AO 31-M/2.1III, 31-M/3.1III, 31-M 3.2III
- Kinder < 6 bis 8 Jahre

## Kontraindikationen

- Trümmerfrakturen ohne Abstützung
- Schenkelhalsfrakturen

## Relative Kontraindikationen

- Gleichzeitige Femurschaftfraktur
- Offene Verletzungen
- Gefäß‑/Nervenverletzungen

## Patientenaufklärung

- Allgemeine Operationsrisiken: Infektion, Blutung, Gefäß‑/Nervenverletzungen, Narkoserisiko
- Mögliche Irritationen der Weichteile durch Nagelenden
- Mangelnde Stabilität mit Schmerzen
- Sekundäre Dislokation

## Operationsvorbereitungen

- Röntgen: Beckenübersicht, Hüfte axial
- Kontrolle des Operationsgebietes: Haut, Weichteile
- Kontrolle und Dokumentation der Neurologie und Durchblutung
- Ausmessen des Femurmarkraums zur Bestimmung der Nageldicke (Ziel 25–30 % des Markraumdurchmessers, da 3 Nägel eingebracht werden müssen)
- Bestimmen und Dokumentieren der Rotation der Hüfte der unverletzten Seite zum späteren, intraoperativen Ausschluss von Rotationsfehlern
- Single-Shot-Antibiose, gewichtsadaptiert

## Instrumentarium

**Figure 1:**
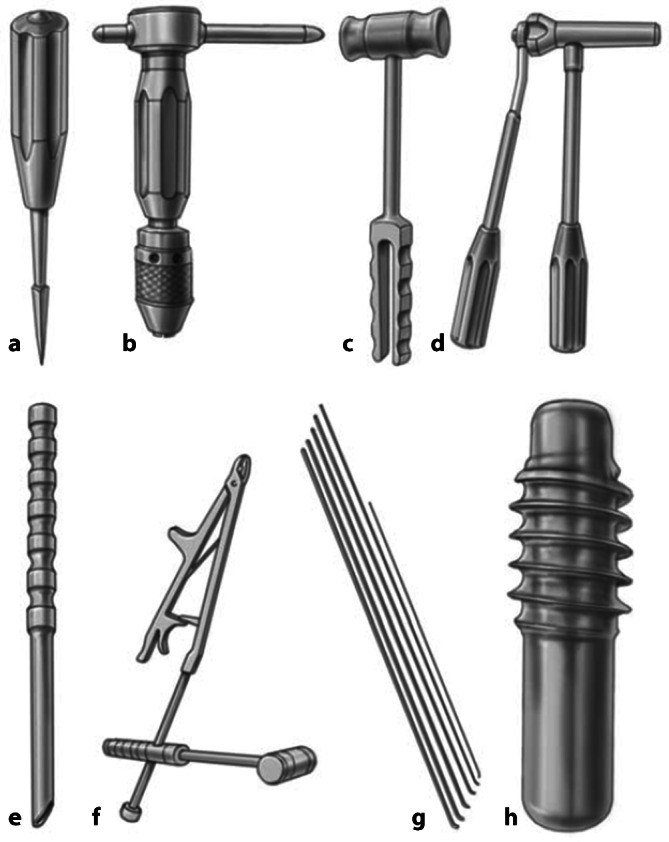

- Bildwandler zum intraoperativen Röntgen
- Standard-ESIN-Instrumentarium (Abb. 1) mit:
  - Aale (Abb. 1a),
  - Handgriff (Abb. 1b),
  - Hammer (Abb. 1c),
  - Nagelschneider oder Bolzenschneider (Abb. 1d),
  - Einschlaginstrument (Abb. 1e),
  - optional Rückschlaginstrumentarium (Abb. 1f),
  - TEN Durchmesser 2,5–3,5 (Abb. 1g)
  - optional Endkappen (Abb. 1h),

## Anästhesie und Lagerung

**Figure 2:**
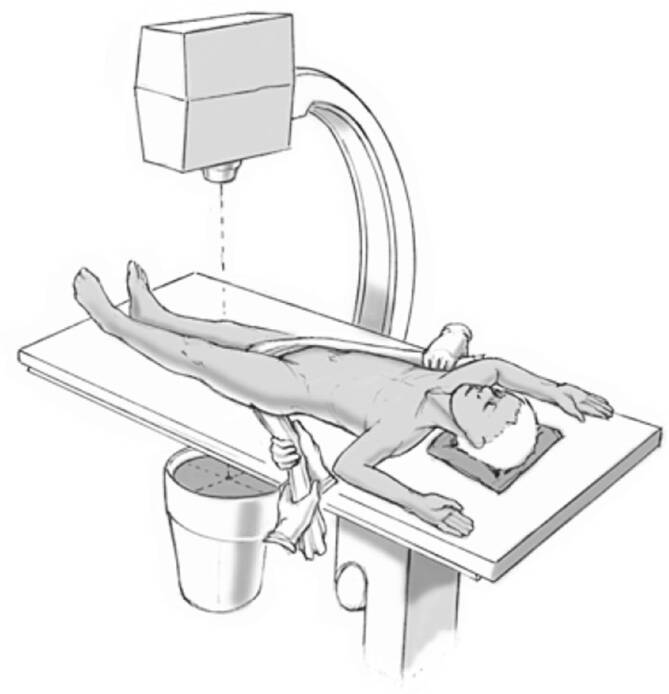

- Allgemeinanästhesie mit Intubation und Muskelrelaxation sind notwendige Voraussetzungen, um eine erfolgreiche geschlossene Reposition zu erreichen.
- Rückenlage
- Röntgendurchlässiger Tisch
- Um einen Gegenzug zu erhalten, wird ein Tuch proximal am Operationstisch befestigt, über die Leiste des gesunden Beines gelegt, anschließend unter dem verletzten Bein hindurchgeführt und auf der Gegenseite proximal am Operationstisch befestigt (Abb. 2).
- Das verletzte Bein wird frei beweglich gelagert und abgedeckt.
- Der Bildwandler wird senkrecht zum Operationstisch von der Gegenseite hereingefahren.

## Operationstechnik

**Figure 3:**
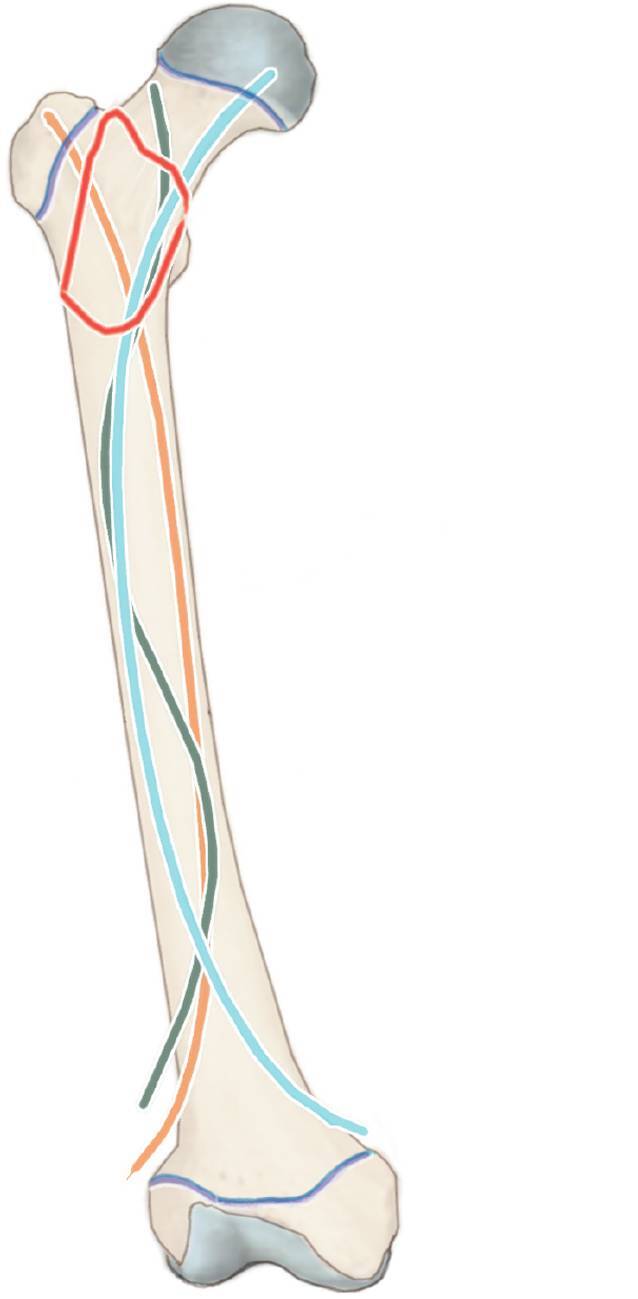

**Figure 4:**
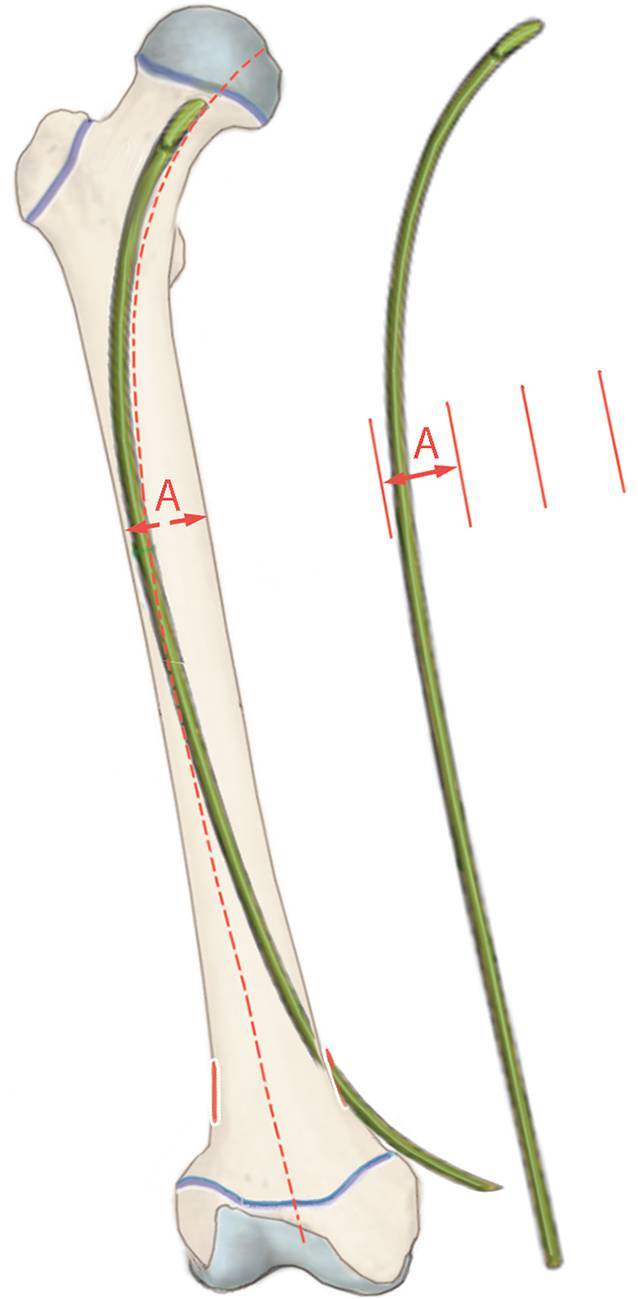

**Figure 5:**
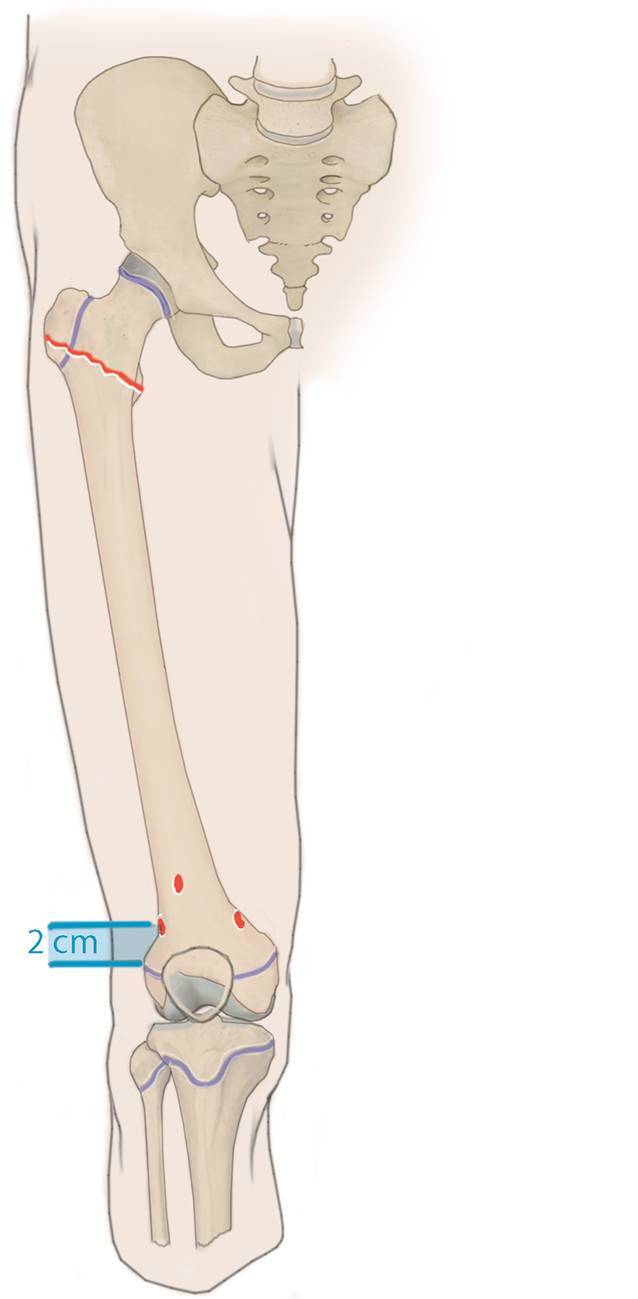

**Figure 6:**
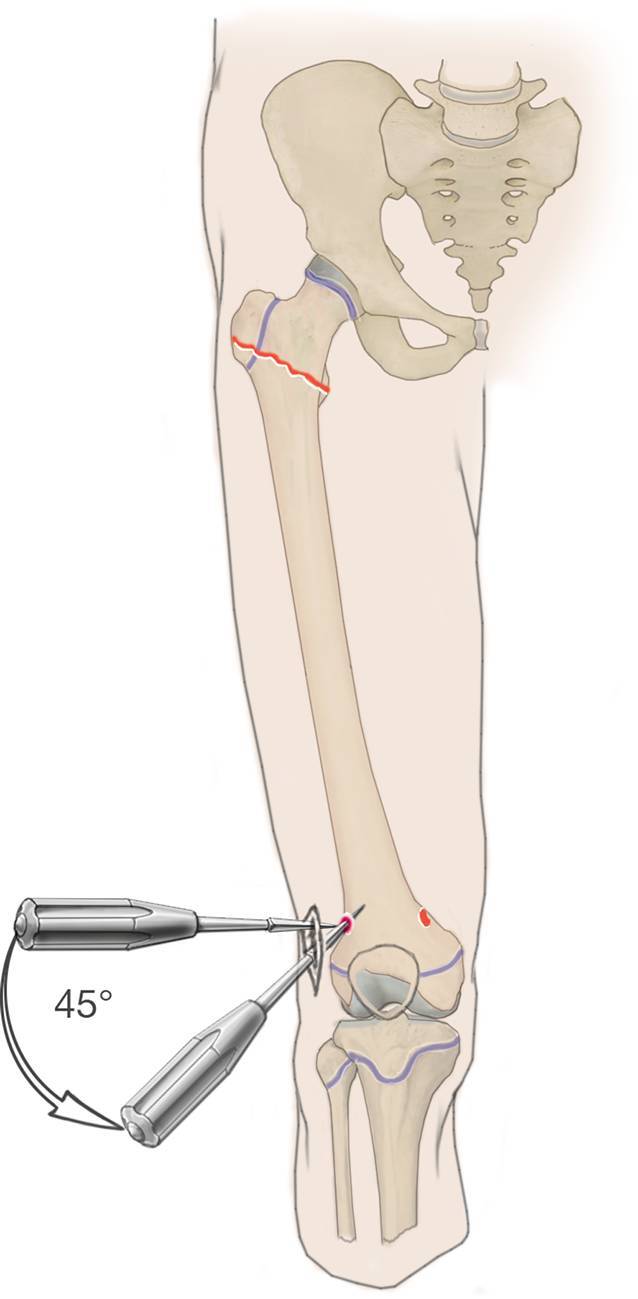

**Figure 7:**
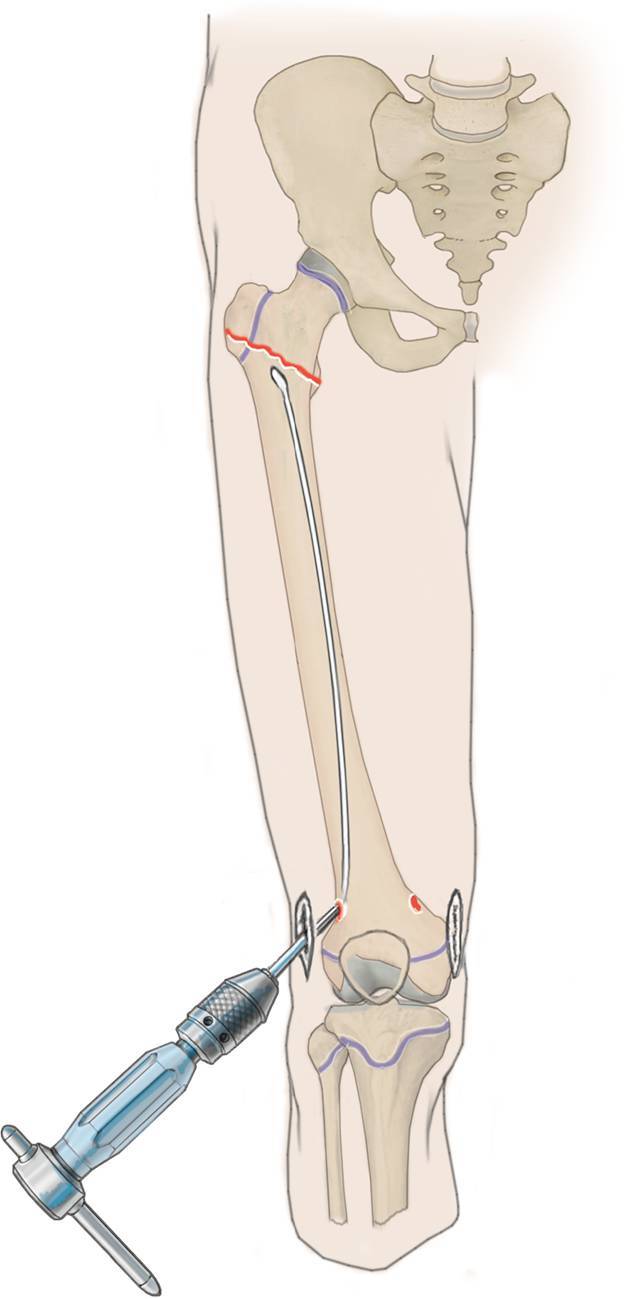

**Figure 8:**
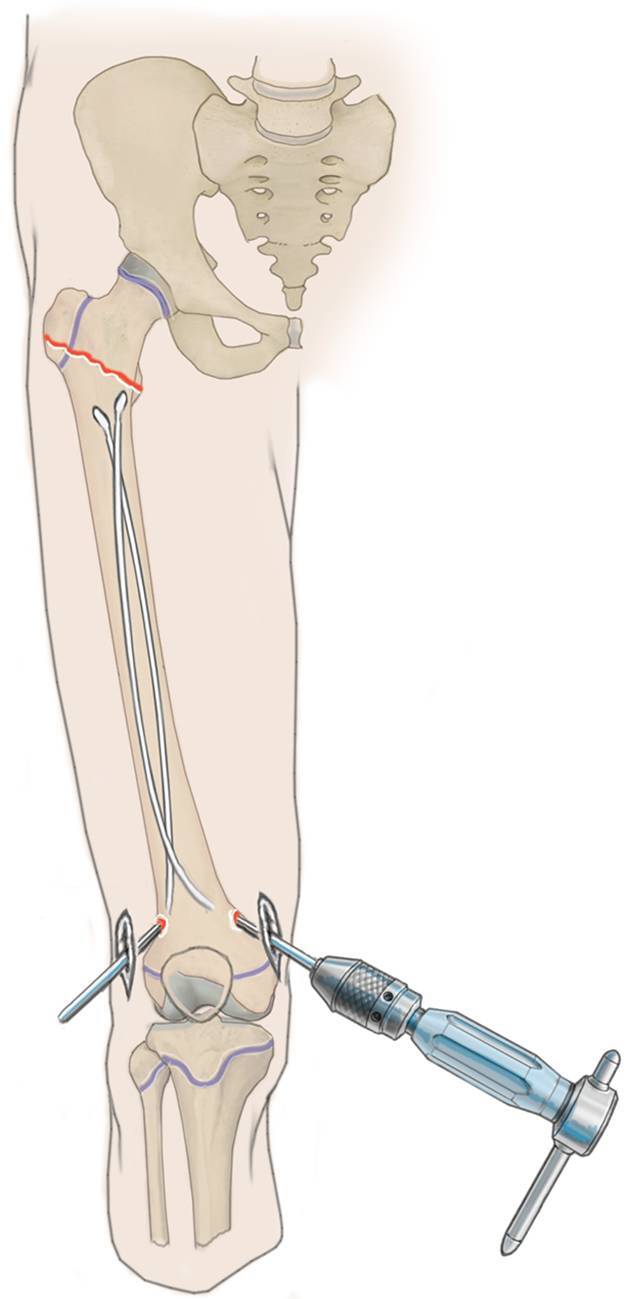

(Abb. 3, 4, 5, 6, 7, 8, 9 und 10).

**Figure 9:**
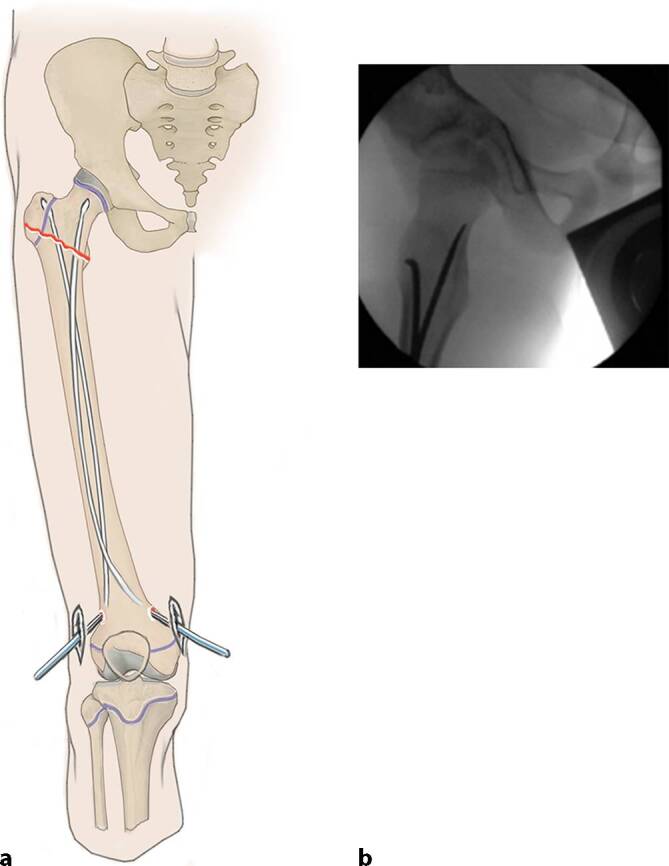

**Figure 10:**
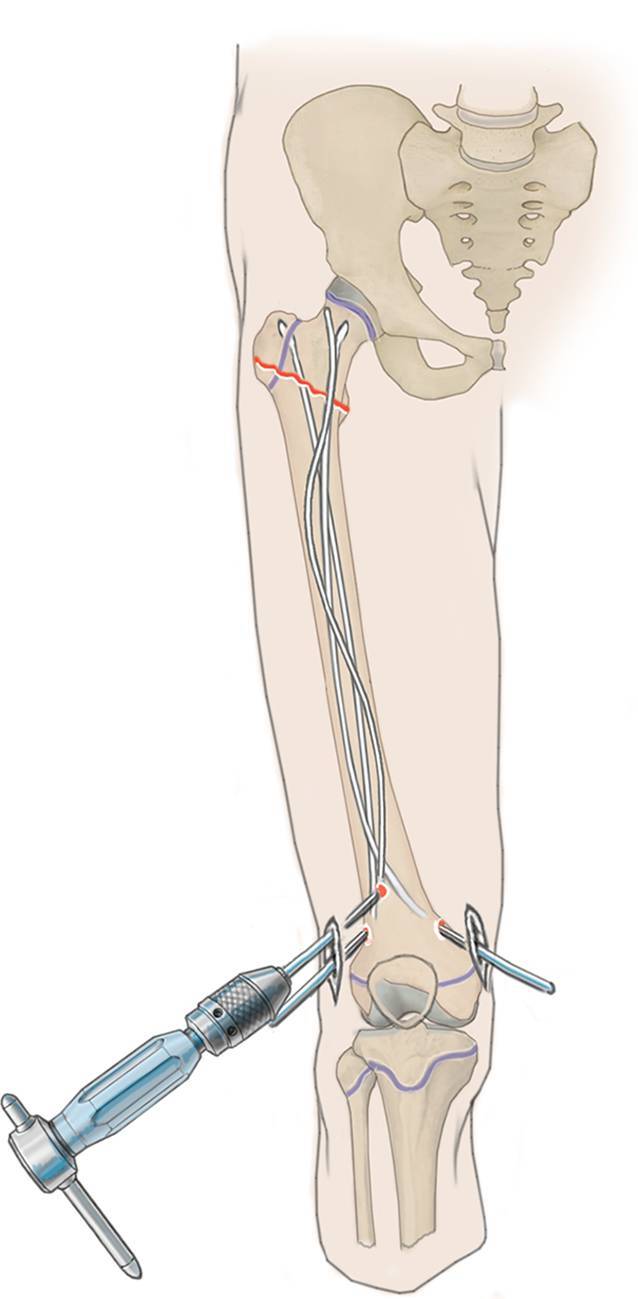

## Fallbeispiel

**Figure 11:**
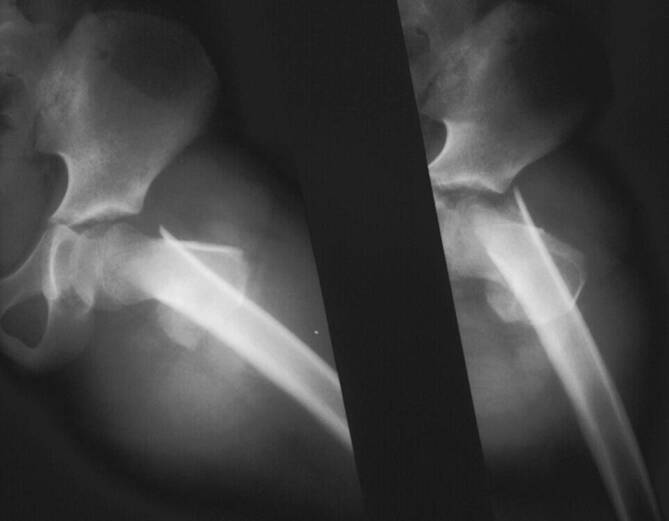

**Figure 12:**
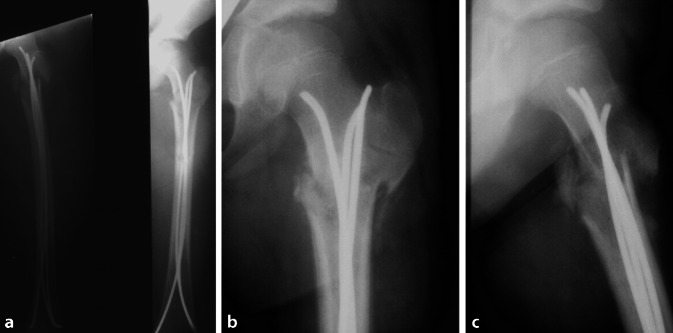

(Abb. 11, 12, 13 und 14).

**Figure 13:**
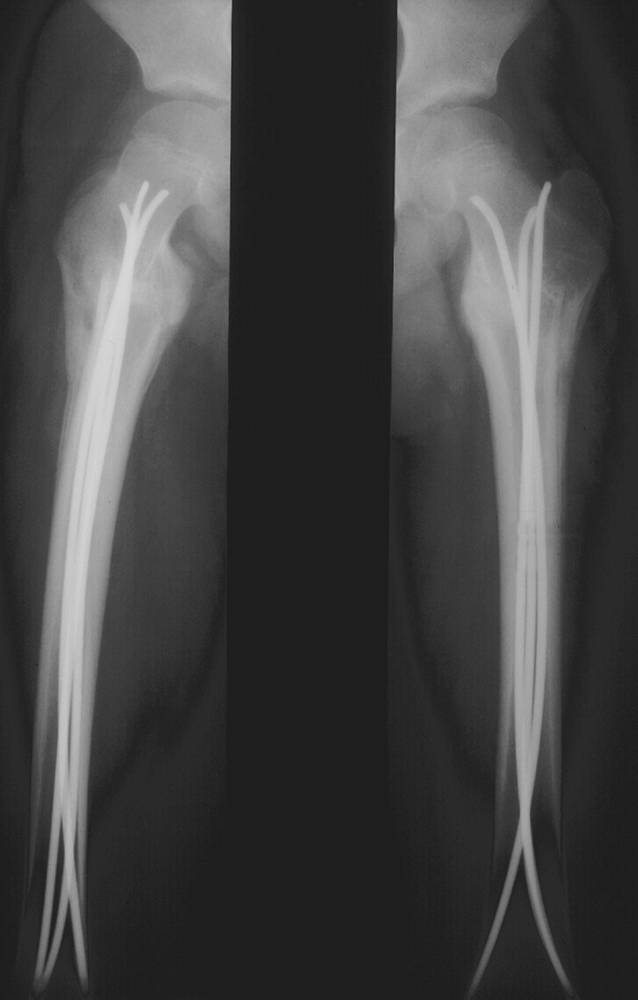

**Figure 14:**
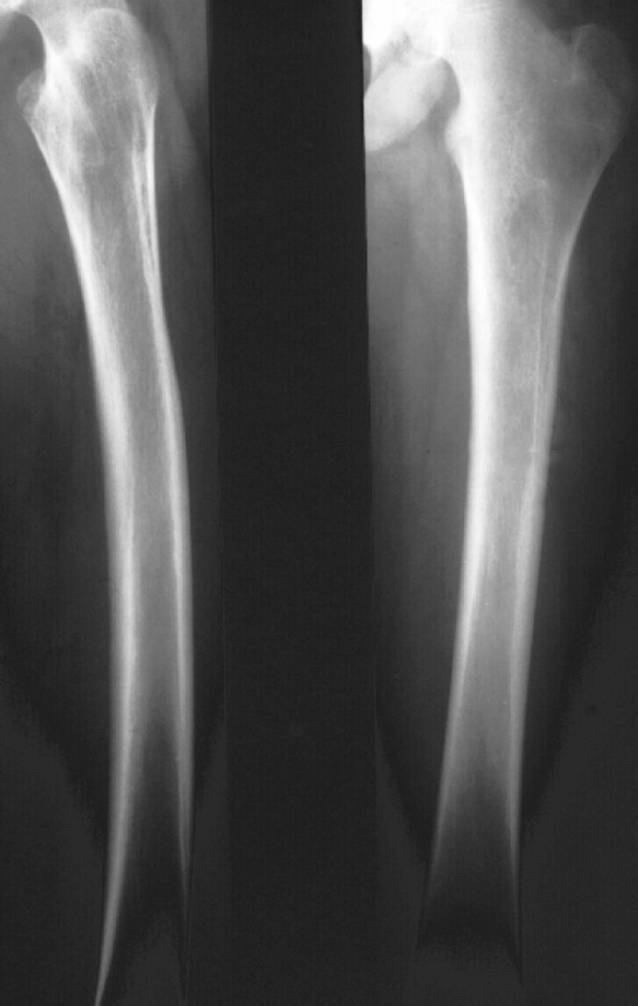

## Besonderheiten

(Abb. 15 und 16).

**Figure 15:**
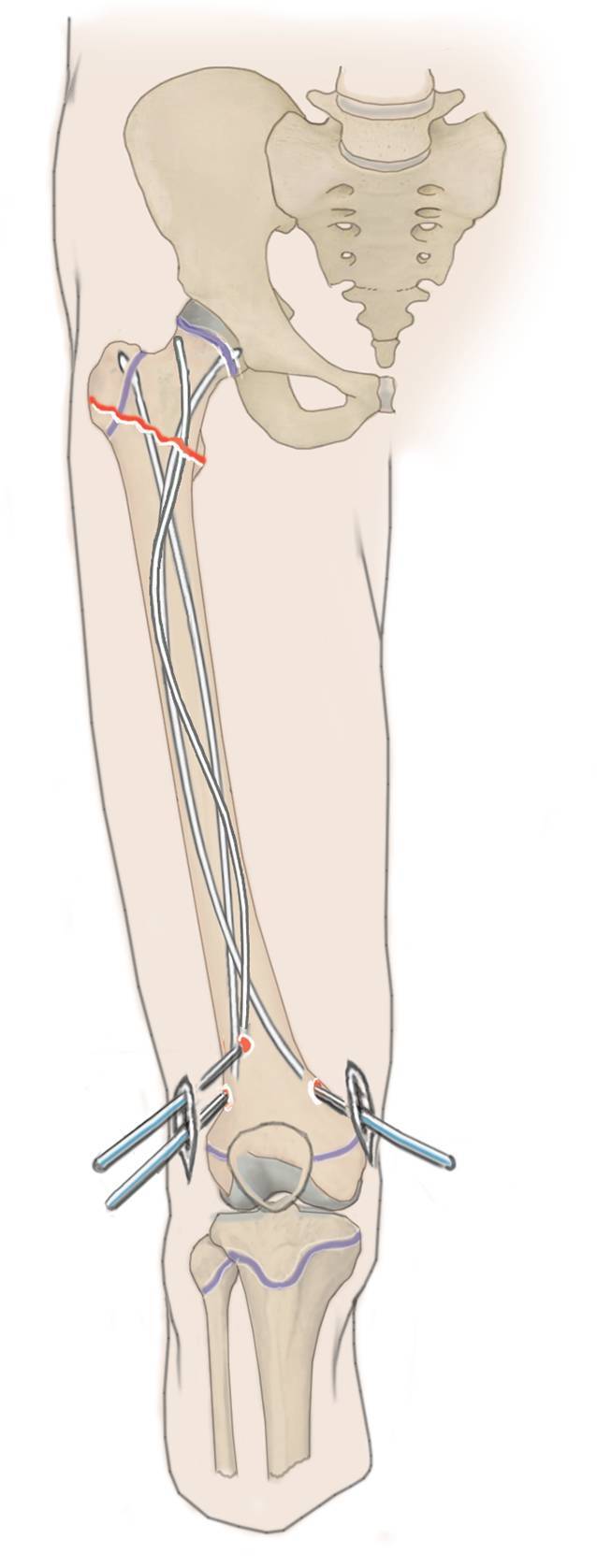

**Figure 16:**
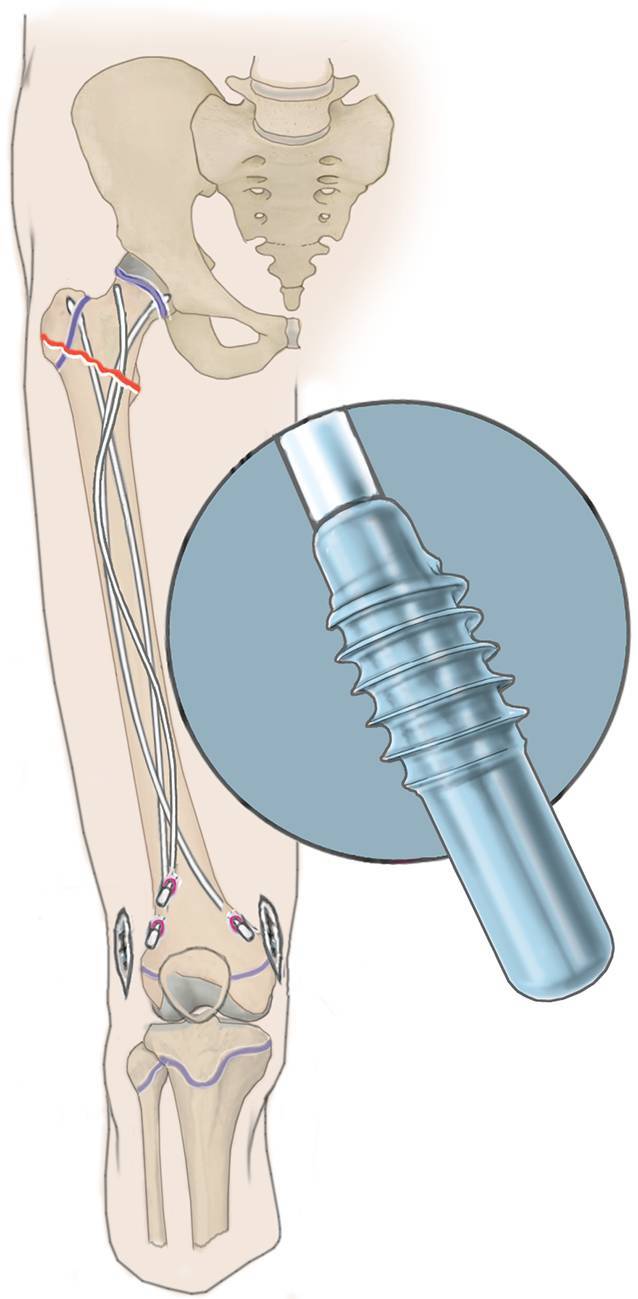

## Postoperative Behandlung

- Radiologische Kontrolle (Becken a.-p. und Hüfte axial) postoperativ sowie nach 4 bis 5 Wochen und nach 10 bis 12 Wochen
- Mobilisation mit Sohlenbelastung (5 kg) für 4 bis 5 Wochen möglich, sofern das Kind in diesem Alter dazu fähig ist, ansonsten mit einem Transfer Bett-Rollstuhl
- Bei korrekter radiologischer Kontrolle nach 4 bis 5 Wochen schmerzadaptierter Übergang zur Vollbelastung
- Kindergarten/Schulunfähigkeit für ca. 2 Wochen je nach Regeln des jeweiligen Schulbetriebes
- Sportunfähigkeit für 3 Monate (keine Kontaktsportarten während 6 Monaten)
- Nagelentfernung nach vollständiger Konsolidation der Fraktur, in der Regel nach 5 bis 6 Monaten

## Fehler, Gefahren, Komplikationen und ihre Behandlung

- Kortikalisperforation am Kalkar oder Trochanter: Neuorientieren der Nagelspitzen
- Geschlossene Reposition nicht möglich: offene Reposition über kleinen lateralen Zugang zum proximalen Femur
- Übungsstabile 3‑Punkte-Abstützung wird nicht erreicht: Verbesserung der Stabilität durch Endcaps oder Anlage eines Becken-Bein-Gipses
- Reizung durch Nagelenden mit Pseudobursa: Kürzen der Nägel oder frühzeitige Nagelentfernung anstreben
- Sekundäre Dislokation (in der Regel in den Varus): Reosteosynthese, ggf. mit Verfahrenswechsel (pädiatrische Hüftplatte)

## Ergebnisse

Wie bereits eingangs erwähnt, handelt es sich um sehr seltene Frakturen, insbesondere in der beschriebenen Altersklasse < 6 bis 8 Jahre. Die hier beschriebene Technik bietet eine weitere, wenig invasive Möglichkeit der Versorgung dieser Frakturen bei kleineren Kindern durch den in der Technik der ESIN erfahrenen Operateur. Eine statistisch korrekte, systematische Zusammenfassung der Ergebnisse ist jedoch aufgrund der naturgemäß geringen Fallzahl nicht möglich.
